# Supplementary material for: Double-strand break repair pathways differentially affect processing and transduction by dual AAV vectors
Source: Nat Commun. 2025 Feb 11;16:1532. doi: 10.1038/s41467-025-56738-5 (PMC11814140; doi:10.1038/s41467-025-56738-5)
Supplement: Supplementary file 1 — Supplementary Figs. [file 41467_2025_56738_MOESM1_ESM.pdf]

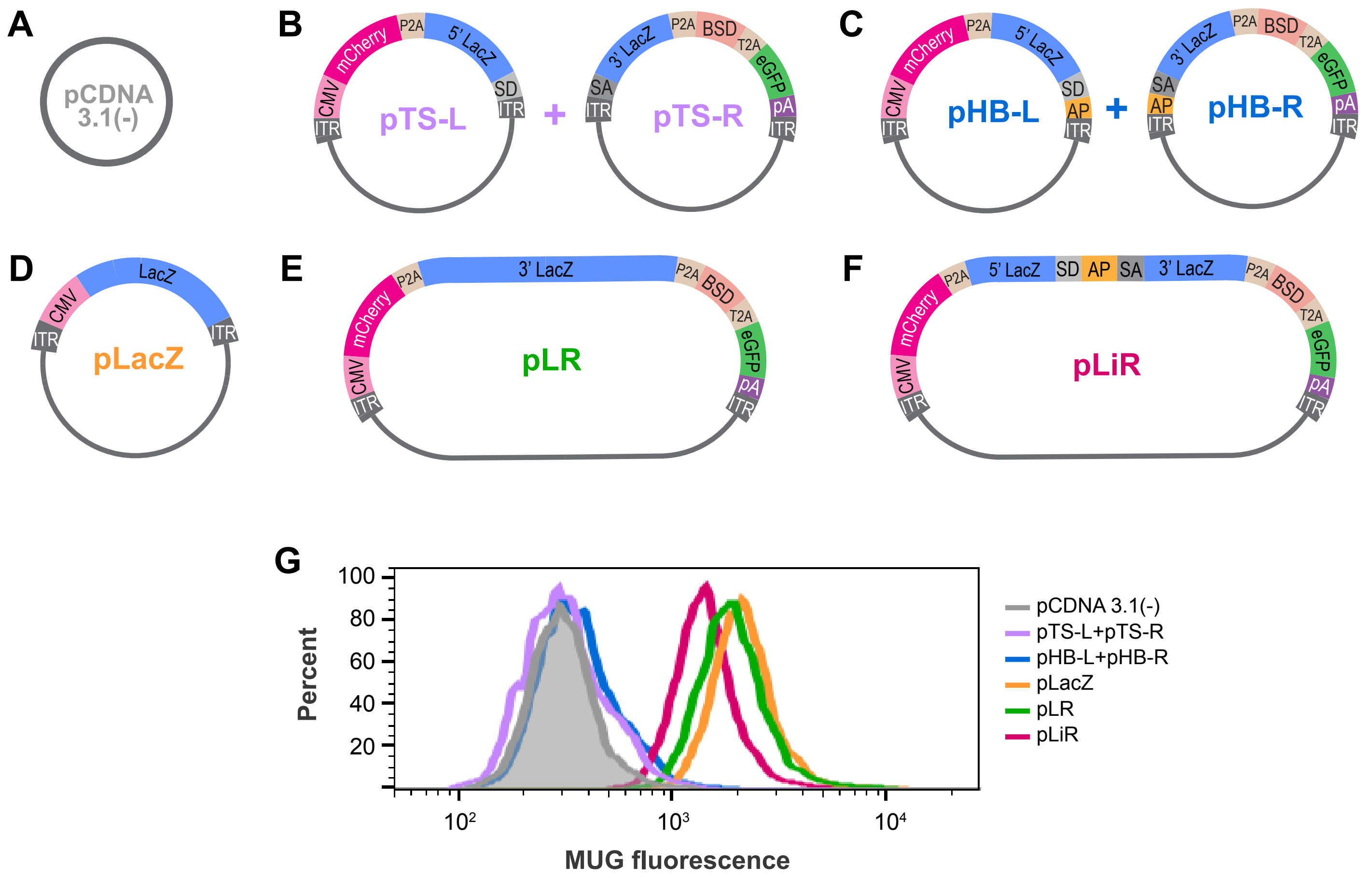

Supplementary Figure 1. Dual vector controls. (A) An empty pCDNA 3.1(-) plasmid served as a negative control. (B) Plasmids containing the Trans-Splicing (TS) Left (L) and Right (R) vector genomes, (C) the Hybrid (HB) L and R vector genomes, (D) the unsplit LacZ vector genome, (E) an oversized vector genome that recapitulates a spliced version of concatenated TS and HB pairs, (F) an oversized vector genome recapitulating the HB pair after recombination at the AP fragment in backbones with an ampicilin resistance cassette and a bacterial ori were co-transfected. (G) 48 hours after transfection, cells were colected and LacZ expression measured by LAFA and flow cytometry. The histogram represents 4-Methylumbelliferyl α-D-galactoside (MUG) fluorescence in 5000 cells per condition.

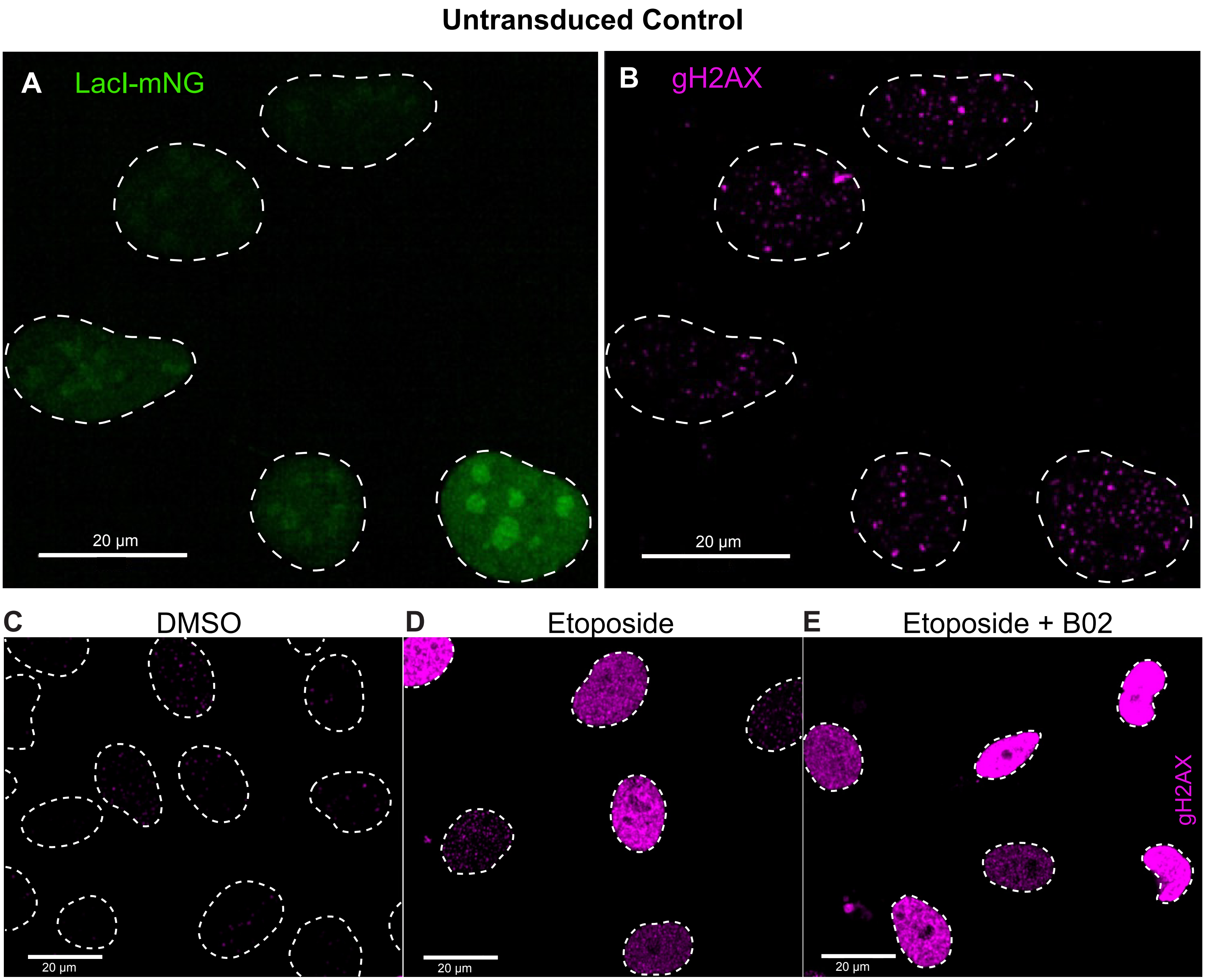

Supplementary Figure 2. Untransduced and DNA damage staining controls. (A, B) U2OS$^{LacI\text{-}mNeonGreen}$ cells were fixed and immunostained for gamma-H2AX. A & B are representative of 6 biological replicates. U2OS$^{LacI\text{-}mNeonGreen}$ cells were treated with **(C)** DMSO, **(D)** 10 µM Etoposide, or **(E)** 10 µM Etoposide and 10 µM B02, then fixed at 36h and immunostained for gH2AX. C, D, & E images are representative of experiments in biological duplicate. DMSO = dimethyl sulfoxide.

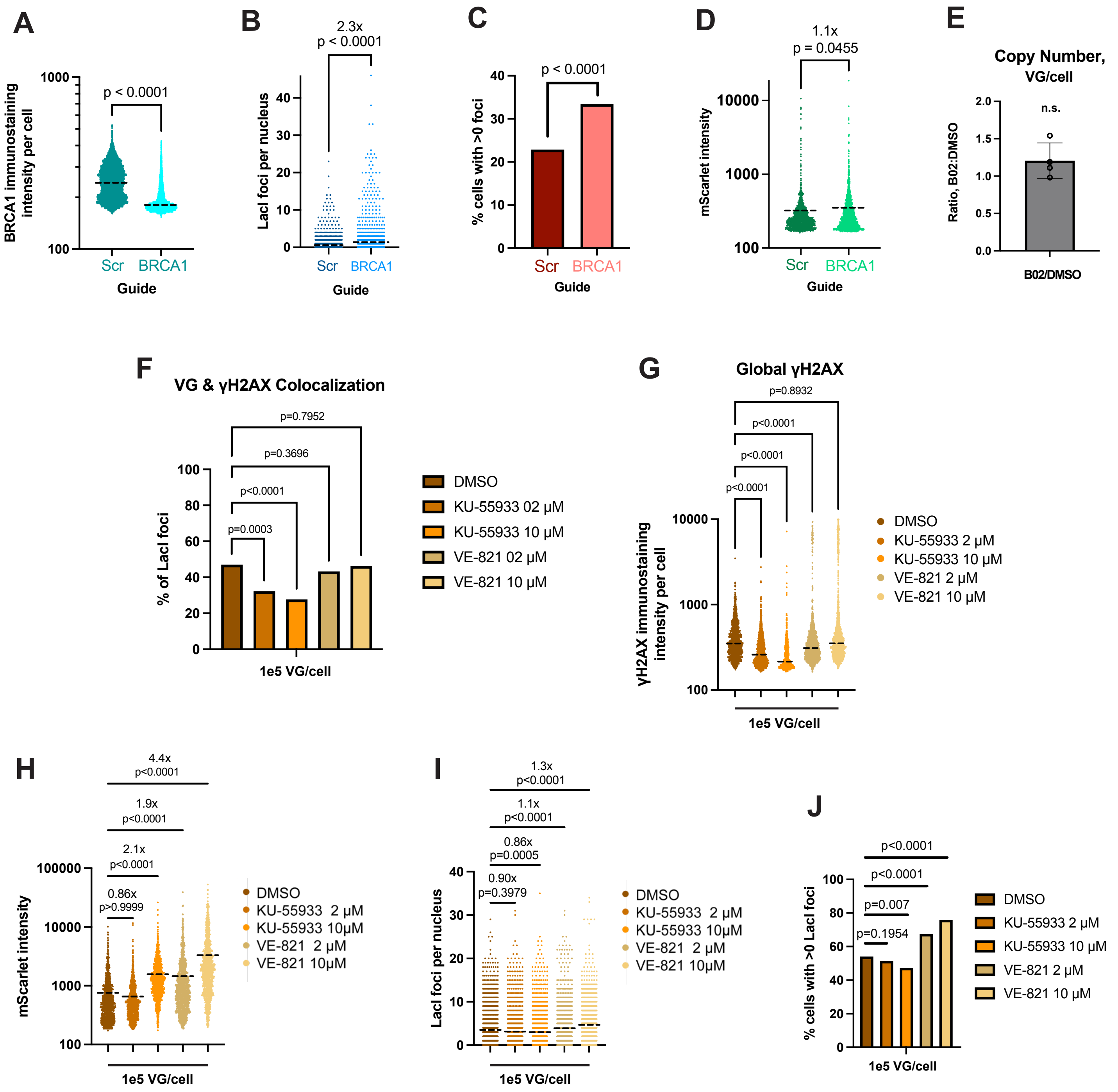

Supplementary Figure 3. Effects of BRCA1, ATM, and ATR perturbations on transduction. U2-OS[LacI-mNeonGreen] cells stably expressing Cas9 were transfected with BRCA1 or Scramble (Scr) guide expressing plasmids, placed under selection for 72h, then transduced with 1e5 vg/cell of AAV2/2.lacO.CMV.mScarlet and high-content imaged at 48 hpt. Cells were immunostained and individual cells are plotted as points by (A) BRCA1 immunostaining intensity, (B) number of LacI foci, (C) the percentage of cells with any LacI foci, and (D) transgene expression intensity. For A-D, n=1490 cells per condition; plots are representative of similar results obtained in biological triplicate. (E) Vector copy number per cell determined by qPCR 48 hpt with 1e5 vg/cell, in DMSO and B02 treated cells. n=4 biological replicate wells are plotted as individual points as the ratio of B02:DMSO copy numbers. (F-J) Cells were pretreated for 8h with the indicated drug, transduced with the indicated vector dose, fixed at 48 hpt, and high-content imaged (n=1303 cells per condition; plots are representative of similar results obtained in biological triplicate). KU-55933: ATM inhibitor. VE-821: ATR inhibitor. DMSO: dimethyl sulfoxide. (F,G) Cells were immunostained for gH2AX, and in (F) LacI foci whose center overlapped with a γH2AX focus were quantified and plotted as a percentage of all foci imaged. (G) Cells are plotted by nuclear immunostaining intensity. (I) Individual cells are plotted according to quantified LacI foci. (J) The perecentage of all imaged cells with any LacI foci was quantified. (H) Transgene expression was quantified by mScarlet intensity and cells were plotted as individual points.
Source data for plots are provided as a Source Data File. Black dotted lines = mean value; fold change between means indicated as numbers above graphs. Error bars = SD. Statistical significance was determined by Kruskal-Wallis Test with Dunn's multiple comparisons test in (A, B, D, G, H) and (I), by paired, two-tailed T test in (E), by Fisher's exact test in (C & J) and ANOVA in (F), with p values for each comparison indicated above black bars. VG = vector genome.

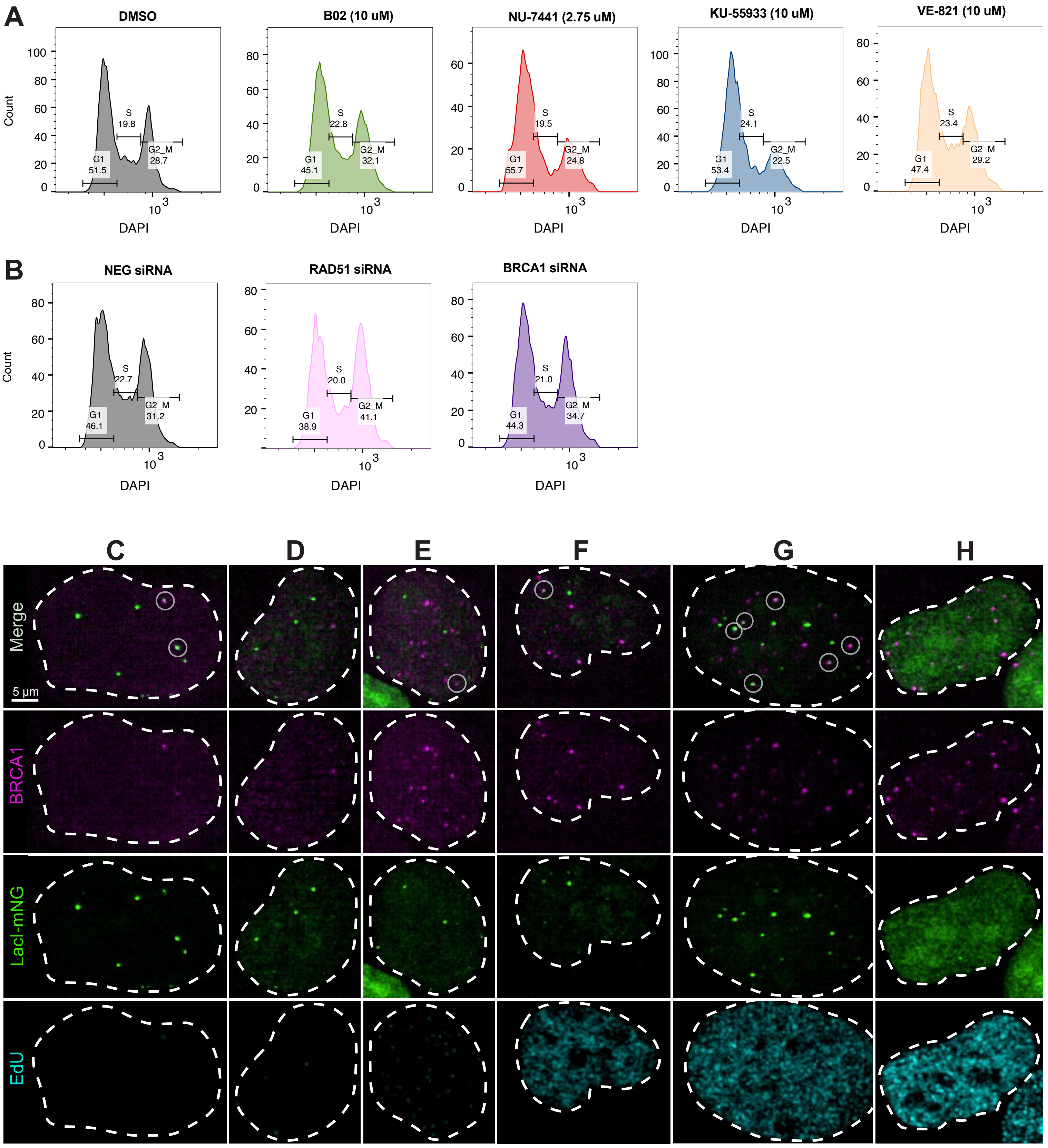

Supplementary Figure 4. Cell Cycle Effects are Minimal. (A) U2-OS$^{LacI\text{-}mNeonGreen}$ cells were treated with drug listed above plot for 40h, then DNA content was measured by DAPI staining and flow cytometry on 5000 cells. Cell cycle phases were gated on dimethyl sulfoxide (DMSO) treated cells and applied to all panels. Numbers indicate percentage of cells in indicated phase. (B) U2-OS$^{LacI\text{-}mNeonGreen}$ cells were transfected with the indicated siRNA, then processed as in (A) at 40h post-transfection. (C-H) U2-OS$^{LacI\text{-}mNeonGreen}$ cells were transduced with AAV2/2.laco.64.CMV.mScarlet at 1e5 VG/cell. At 48 hpt, cells were pulsed with 5-Ethynyl-2-deoxyuridine (EdU) for 15 min before fixation. EdU was detected with 647-Azide and cells were immunostained for BRCA1, then high-content imaged. Representative cells were selected ouside of S-phase (C-E) and in S-phase (F-H), and with various numbers of LacI foci. Grey circles indicate BRCA1 and LacI-mNeonGreen (mNG) colocalization. Images are representative of experiments in biological duplicate.

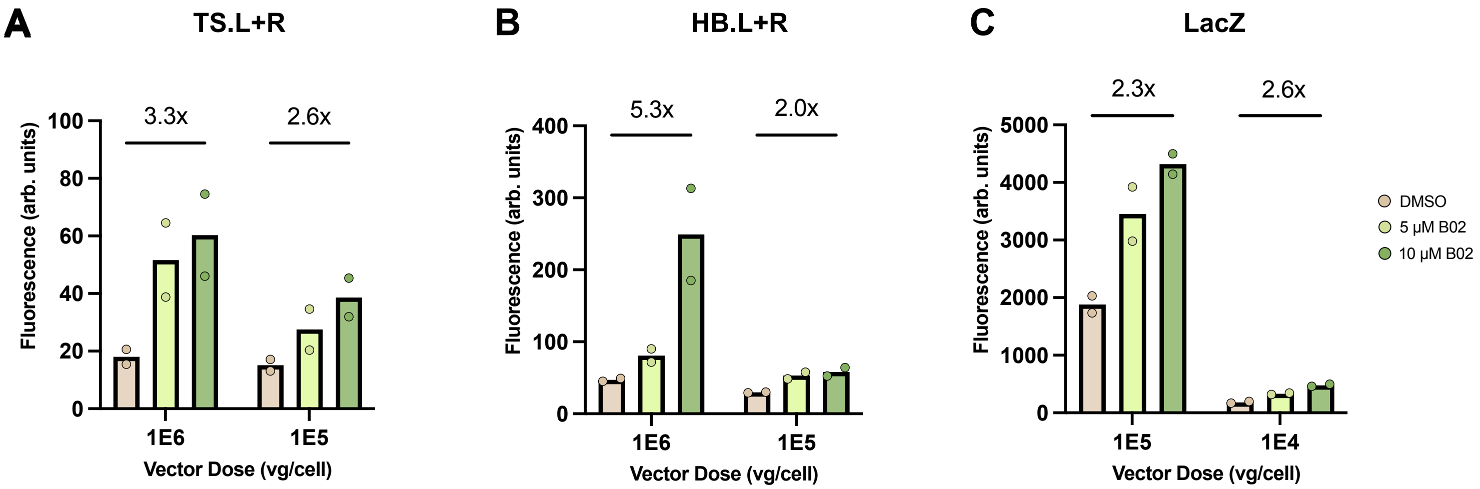

Supplementary Figure 5. Rad51 inhibition increases dual vector transduction in Trophoblast cells. The effects of Rad51 inhibition on transduction were tested in BeWo cells by LAFA 48 hours post-trasduction by (A) TS dual vectors, (B) HB dual vectors, and (C) LacZ single vectors at doses on x-axis. Bars represent the mean of 2 individually plotted biological replicates.. Numbers above charts indicate fold increase of 10 µM B02 condition compared to dimethyl sulfoxide (DMSO) control. Source data for plots are provided as a Source Data File.
