# Supplementary material for: Double-strand break repair pathways differentially affect processing and transduction by dual AAV vectors
Source: Nat Commun. 2025 Feb 11;16:1532. doi: 10.1038/s41467-025-56738-5 (PMC11814140; doi:10.1038/s41467-025-56738-5)
Supplement: Supplementary file 2 — Description of Additional Supplementary Files [file 41467_2025_56738_MOESM2_ESM.pdf]

**Supplementary Data 1. Table of enriched genes.** The most significant 5% of positive regulators (i.e., genes that, when perturbed, have increased dual vector transduction) were submitted as a gene list for Reactome pathway analysis. Genes grouped by their Reactome pathway(s) are tabulated here with their fold-enrichment scores and p-values.

**Supplementary Data 2. Code used to generate tables and plots of screen data.** Custom code used to generate the table in Supplementary Data 1 and plots in Figure 2b-d is provided as a Jupyter notebook and is also available at https://doi.org/10.5061/dryad.7h44j1053

**Supplementary Movies 1-6. Visualization of VG concatenation in living cells.** U2-OS$^{\text{LacI-mNeonGreen}}$ cells were pretreated with 10 μM B02 (Movies 1-3) or DMSO (Movies 4-6) for 8h, then transduced with 1e6 VG/cell AAV2/2.lacO.CMV.mScarlet + 10 μM B02 or DMSO, respectively. LacI-mNeonGreen fluorescence was imaged every 6 minutes from 4-48 hours post transduction.
