# Supplementary material for: Double-strand break repair pathways differentially affect processing and transduction by dual AAV vectors
Source: Nat Commun. 2025 Feb 11;16:1532. doi: 10.1038/s41467-025-56738-5 (PMC11814140; doi:10.1038/s41467-025-56738-5)
Supplement: Supplementary file 11 — Reporting Summary [file 41467_2025_56738_MOESM11_ESM.pdf]

nature portfolio

Corresponding author(s): Maurer, AC

Last updated by author(s): Jan 20, 2025

Reporting Summary

Nature Portfolio wishes to improve the reproducibility of the work that we publish. This form provides structure for consistency and transparency in reporting. For further information on Nature Portfolio policies, see our Editorial Policies and the Editorial Policy Checklist.

Statistics

For all statistical analyses, confirm that the following items are present in the figure legend, table legend, main text, or Methods section.

n/a	Confirmed	
☐	☒	The exact sample size (*n*) for each experimental group/condition, given as a discrete number and unit of measurement
☐	☒	A statement on whether measurements were taken from distinct samples or whether the same sample was measured repeatedly
☐	☒	The statistical test(s) used AND whether they are one- or two-sided<br>*Only common tests should be described solely by name; describe more complex techniques in the Methods section.*
☐	☒	A description of all covariates tested
☐	☒	A description of any assumptions or corrections, such as tests of normality and adjustment for multiple comparisons
☐	☒	A full description of the statistical parameters including central tendency (e.g. means) or other basic estimates (e.g. regression coefficient) AND variation (e.g. standard deviation) or associated estimates of uncertainty (e.g. confidence intervals)
☐	☒	For null hypothesis testing, the test statistic (e.g. *F*, *t*, *r*) with confidence intervals, effect sizes, degrees of freedom and *P* value noted<br>*Give P values as exact values whenever suitable.*
☒	☐	For Bayesian analysis, information on the choice of priors and Markov chain Monte Carlo settings
☒	☐	For hierarchical and complex designs, identification of the appropriate level for tests and full reporting of outcomes
☒	☐	Estimates of effect sizes (e.g. Cohen's *d*, Pearson's *r*), indicating how they were calculated

Our web collection on statistics for biologists contains articles on many of the points above.

Software and code

Policy information about availability of computer code

Data collection	Data were collected with Harmony (v 5.1), NIS Elements (v 9.0), and BD FACSDiva (v 9.0).
Data analysis	Data were analyzed using the following software: Harmony (v 5.1), FlowJo (v 10.9.0), Graphpad Prism (v 10.1.0), ImageJ (v 2.14.0), and the Broad GPP Screen Analysis Tools available at https://portals.broadinstitute.org/gpp/public/.

For manuscripts utilizing custom algorithms or software that are central to the research but not yet described in published literature, software must be made available to editors and reviewers. We strongly encourage code deposition in a community repository (e.g. GitHub). See the Nature Portfolio guidelines for submitting code & software for further information.

Data

Policy information about availability of data

All manuscripts must include a data availability statement. This statement should provide the following information, where applicable:

- Accession codes, unique identifiers, or web links for publicly available datasets
- A description of any restrictions on data availability
- For clinical datasets or third party data, please ensure that the statement adheres to our policy

Raw sequencing data from the genome-wide screen are available at NCBI Sequencing Read Archive under BioSample accession number PRJNA1195613 [https://www.ncbi.nlm.nih.gov/sra/PRJNA1195613]. Source data for all other figures are provided as a source data file.

Research involving human participants, their data, or biological material

Policy information about studies with human participants or human data. See also policy information about sex, gender (identity/presentation), and sexual orientation and race, ethnicity and racism.

Reporting on sex and gender	N/A
Reporting on race, ethnicity, or other socially relevant groupings	N/A
Population characteristics	N/A
Recruitment	N/A
Ethics oversight	N/A

Note that full information on the approval of the study protocol must also be provided in the manuscript.

Field-specific reporting

Please select the one below that is the best fit for your research. If you are not sure, read the appropriate sections before making your selection.

☒ Life sciences ☐ Behavioural & social sciences ☐ Ecological, evolutionary & environmental sciences

For a reference copy of the document with all sections, see nature.com/documents/nr-reporting-summary-flat.pdf

Life sciences study design

All studies must disclose on these points even when the disclosure is negative.

Sample size	For high-content imaging experiments, sample size was determined based on imaging time (maximum 6 hours) and file size (max 200GB) limitations per biological replicate (at least 2 when results were highly repeatable). The minimum number of imaged cells with these limitations was 530 per condition – well above the generally accepted minimum of 30 cells to generate high statistical power.<br>For experiments with cells in bulk/well format, minimum sample sizes were determined such that a p value can be calculated to be 0.05 or less (n = at least 3). For Figure S5, sample size is n=2 due to labour limitations, but high repeatability of these replicates provides acceptable confidence.<br>Relevant citation for these choices: Kristen Naegle et al., Criteria for biological reproducibility: What does “n” mean?. Sci. Signal. 8, fs7-fs7 (2015). DOI:10.1126/scisignal.aab1125
Data exclusions	There are no data excluded from the manuscript.
Replication	All experiments were performed a minimum of 2 times with highly similar results.
Randomization	Randomization is not relevant to this study, as no human or animal subjects were used.
Blinding	Blinding is not relevant to this study, as no human or animal subjects were used.

Reporting for specific materials, systems and methods

We require information from authors about some types of materials, experimental systems and methods used in many studies. Here, indicate whether each material, system or method listed is relevant to your study. If you are not sure if a list item applies to your research, read the appropriate section before selecting a response.

Materials & experimental systems

n/a	Involved in the study	
☐	☒	Antibodies
☐	☒	Eukaryotic cell lines
☒	☐	Palaeontology and archaeology
☒	☐	Animals and other organisms
☒	☐	Clinical data
☒	☐	Dual use research of concern
☒	☐	Plants

Methods

n/a	Involved in the study	
☒	☐	ChIP-seq
☐	☒	Flow cytometry
☒	☐	MRI-based neuroimaging

Antibodies

Antibodies used	Immunofluorescence:<br>Rb anti-53BP1: Abcam ab175188, lot# GR135905-4, 1:10,000. Rb Phospho-Histone H2A.X: Cell Signaling Technology 25775, lot# 14, 1:2500. Ms anti-BRCA1: Santa Cruz sc-6954, lot# J1821, 1:2000. Rb anti-Rad51: Axxora CAC-BAM-70-001-EX, lot# 04, 1:4000, and ab133534, lot# GR3270300-18, 1:2500.<br><br>ChIP-qPCR:<br>Normal rabbit IgGs: Abcam #ab46540, rabbit anti-Phospho-Histone H2A.X (Ser139) (Cell Signaling Technology #2577, lot# 14) and rabbit anti-Histone H2B (Invitrogen # MA5-24697).<br><br>Western blotting:<br>Rb anti-Rad51: ab133534, lot# GR3270300-18, 1:2000. Ms anti-BRCA1: Santa Cruz sc-6954, lot# J1821, 1:1000. Ms anti-TBP: Recombinant Anti-TATA binding protein TBP antibody [mAbcam51841] (ab300656), lot# GR135905-4, 1:2500.
Validation	All antibodies were validated previously in the literature and by the manufacturers:<br>53BP1: Han J, Ruan C, Huen MSY, Wang J, Xie A, Fu C, Liu T, Huang J. BRCA2 antagonizes classical and alternative nonhomologous end-joining to prevent gross genomic instability. Nat Commun. 2017 Nov 13;8(1):1470. doi: 10.1038/s41467-017-01759-y. PMID: 29133916; PMCID: PMC5684403. (Figure 7)<br>yH2AX: https://www.cellsignal.com/products/primary-antibodies/phospho-histone-h2a-x-ser139-20e3-rabbit-mab/9718?utm_term&utm_campaign=SO-Products-PrimaryAntibodies&utm_source=adwords&utm_medium=ppc&hsa_acc=8625036580&hsa_cam=21865491299&hsa_grp=167649643577&hsa_ad=719485262778&hsa_src=g&hsa_tgt=dsa-2373170399499&hsa_kw&hsa_mt&hsa_net=adwords&hsa_ver=3&gad_source=1&gclid=Cj0KCQiAhbi8BhDIARIsAJLOlucLYA9sTAjeiRttph_B38buejpYKDMfL9vN3K3aBiTDs_qPRSOzDkoaApdREALw_wcB<br>BRCA1: https://www.scbt.com/p/brca1-antibody-d-9?gad_source=1&gclid=Cj0KCQiAhbi8BhDIARIsAJLOludxvwYUdmjLp5dpU2ftffb8yfxqqfOXMy_GMfzFR7TlBTLNXEoDh_0aAuXMEALw_wcB<br>Rad51: https://www.abcam.com/en-us/products/primary-antibodies/rad51-antibody-epr40303-ab133534?srsltid=AfmBOooZJbrxogiCN0Hkq4_g2yy6kG7RFfw7ME0tlYCXVUR3Of_86c1X<br>H2B: https://www.thermofisher.com/antibody/product/Histone-H2B-Antibody-clone-RM230-Recombinant-Monoclonal/MA5-24697<br>TBP: James ZJ Kwan, Thomas F Nguyen, Anuli C Uzozie, Marek A Budzynski, Jieying Cui, Joseph MC Lee, Filip Van Petegem, Philipp F Lange, Sheila S Teves (2023) RNA Polymerase II transcription independent of TBP in murine embryonic stem cells eLife 12:e83810 (Figure 1).

Eukaryotic cell lines

Policy information about cell lines and Sex and Gender in Research

Cell line source(s)	HEK293T cells used for vector production were obtained from the UC Berkeley BDS Cell Culture Facility (original source ATCC CRL-3216). U2-OS cells were obtained from the UC Berkeley BDS Cell Culture Facility cat# 608)
Authentication	Cell lines are authenticated by the UC Berkeley BDS Cell Culture Facility.
Mycoplasma contamination	Cell lines were tested monthly for mycoplasma by PCR and were negative each time.
Commonly misidentified lines<br>(See ICLAC register)	HEK293T & U2-OS are commonly misidentified lines. HEK293T are the standard line for rAAV production, and the stocks we used produced high-titer vector that passed all quality control. U2-OS cells are commonly used for subcellular imaging experiments because they are very flat and allow for reliable quantification of objects like foci which overlap less frequently in this cellular morphology. Additionally, U2-OS are easily transduced by rAAV vectors. The cell stocks we obtained fit the required criteria and were therefore used in subsequent experiments despite the possibility of misidentification. Results were later validated in additional cell lines.

Plants

Seed stocks	*Report on the source of all seed stocks or other plant material used. If applicable, state the seed stock centre and catalogue number. If plant specimens were collected from the field, describe the collection location, date and sampling procedures.*
Novel plant genotypes	*Describe the methods by which all novel plant genotypes were produced. This includes those generated by transgenic approaches, gene editing, chemical/radiation-based mutagenesis and hybridization. For transgenic lines, describe the transformation method, the number of independent lines analyzed and the generation upon which experiments were performed. For gene-edited lines, describe the editor used, the endogenous sequence targeted for editing, the targeting guide RNA sequence (if applicable) and how the editor was applied.*
Authentication	*Describe any authentication procedures for each seed stock used or novel genotype generated. Describe any experiments used to assess the effect of a mutation and, where applicable, how potential secondary effects (e.g. second site T-DNA insertions, mosiacism, off-target gene editing) were examined.*

Flow Cytometry

Plots

Confirm that:

☒ The axis labels state the marker and fluorochrome used (e.g. CD4-FITC).

☒ The axis scales are clearly visible. Include numbers along axes only for bottom left plot of group (a 'group' is an analysis of identical markers).

☒ All plots are contour plots with outliers or pseudocolor plots.

☒ A numerical value for number of cells or percentage (with statistics) is provided.

Methodology

Sample preparation	cells were seeded in 12-well plates and treated with drug or transfected with siRNA 24h later. At 40 or 36h post treatment, cells were trypsinized and pelleted, resuspended in Phenol Red-free DMEM + 0.01% digitonin + 2.5g/mL DAPI, and 5000 cells per condition were analyzed by flow cytometry
Instrument	LSR Fortessa from BD BIosciences
Software	FACSDiva software (v 9.0) from BD Biosciences.
Cell population abundance	N/A
Gating strategy	Cell cycle phases were gated on dimethyl sulfoxide (DMSO) treated cells based on DAPI staining peaks in the histogram, and applied to all other conditions.

☒ Tick this box to confirm that a figure exemplifying the gating strategy is provided in the Supplementary Information.
